# The Effect of Season-Long Temperature Increases on Rice Cultivars Grown in the Central and Southern Regions of China

**DOI:** 10.3389/fpls.2017.01908

**Published:** 2017-11-06

**Authors:** Zhiyuan Yang, Zuolin Zhang, Tong Zhang, Shah Fahad, Kehui Cui, Lixiao Nie, Shaobing Peng, Jianliang Huang

**Affiliations:** ^1^National Key Laboratory of Crop Genetic Improvement, MOA Key Laboratory of Crop Ecophysiology and Farming System, College of Plant Science and Technology, Huazhong Agricultural University, Wuhan, China; ^2^Department of Agriculture, University of Swabi, Swabi, Pakistan; ^3^Hubei Collaborative Innovation Center for Grain Industry, Yangtze University, Hubei, China

**Keywords:** elevated temperature, indica ecotype, heat-resistant, grain yield, pollen viability

## Abstract

Rice production is challenged by the asymmetric increases in day and night temperatures. Efforts are required to improve our understanding of the impact of climate change on rice production. To this end, 2-year experiment was conducted to evaluate the response of mid-season rice growth in the central and southern regions of China to elevated temperatures. Four replicates of four widely planted indica rice cultivars (Huanghuazhan: HHZ; Shanyou63: SY63; Yangliangyou6: YLY6; Liangyoupeijiu: LYPJ) were subjected to four elevated-temperature treatments (control: ambient temperature; NW: night-time warming; DW: daytime warming; AW: all-day warming) generated by an open-top hot-blast system under field conditions. This apparatus causes an ~2°C increase in the rice canopy temperature. Of all the elevated-temperature treatments, AW was the most devastating treatment for all rice cultivars, negatively affecting nearly all of investigated parameters, including grain yield and its components, dry matter accumulation, biomass, and harvest index (HI). The AW treatment decreased the grain yield by 11–35% and 43–78% in 2015 and 2016, respectively. No significant reduction in the grain yield was observed in the DW and NW treatments in 2015. However, the grain yield was decreased in DW and NW treatments by 20–52% and 18–55%, respectively, in 2016. Furthermore, the temperature-driven degradation of pollen viability, the number of pollen grains adhering to the stigma and pollen germination on the stigma caused spikelet sterility and thereby decreased the grain yield. The YLY6 and SY63 cultivars performed better than the HHZ and LYPJ cultivars with respect to grain yield and its components in all elevated-temperature treatments in both years. However, 42.97 and 61.01% reductions still occurred for the SY63 and YLY6 cultivars, respectively, in the AW treatment in 2016. The above results suggested that the elevated temperature may cause a noteworthy reduction in the productions of these widely planted genotypes in central and southern regions of China. To ensure the security of rice production in this region in an expected global warming environment, currently planted varieties will need to be replaced by heat-resistant varieties in the future.

## Introduction

Global warming poses a threat to the food security and is considered a major challenge in the Twenty-first century to providing enough food for an increasing population in a stressful situation (Lal et al., 2005). Several crops have been exposed to elevated temperatures due to the recent increase in global warming, consecutively influenced a substantial amount of yield rise ascended from several factors. Previous reports have showed that warming might significantly decrease the yield of cereal crops. For example, a predicted 1°C increase in the night-time air temperature might lead to a 10% decrease in rice yield (Peng et al., 2004). The mean surface air temperature has increased globally by ~0.74°C in the last 100 years and will further increased by ~1.1–6.4°C by the end of this century (IPCC, 2007). The extent of the daily maximum temperature increase has been lower than that of the daily minimum temperature, and it is expected to continue (Price et al., 1999; IPCC, 2001; Lobell, 2007). This trend of a decreased diurnal temperature range under future climate (IPCC, 2007) will have increasingly adverse effects on rice.

Studies related to climate effects and adaptation approaches are garnering more interests among scientists worldwide, such as studies on the effects on the crop production e.g., rice, wheat and maize (Hoogenboom, 2000; Gbetibouo and Hassan, 2005; Fahad et al., 2015a,b, 2016a,d, 2017). Flowering has been identified as the stage most sensitive to high-temperature stress, and the prevailing ambient temperatures during anthesis (i.e., flower opening) have been causally linked to reproductive outcomes (Yoshida et al., 1981; Jagadish et al., 2007). Rice flowers open for ~45 min, during which a series of heat stress-sensitive processes occur, such as anther dehiscence (the process through which pollen is released from the anthers), pollination (in which the pollen grains are deposited onto the surface of the stigma (Matsui et al., 2001; Jagadish et al., 2010), pollen germination and pollen tube growth. Fertilization is typically completed within 1.5–4 h after anthesis (Cho, 1956). At temperatures below the maximum for rice, the response of biomass production is the key determinant of yield variations. A variable biomass response to a season-long increase in air temperature has also been reported; the biomass decreased by 16% when the temperature increased from 25 to 27°C (Baker and Allen, 1993). No significant difference in biomass was seen when the temperature increased from 25 to 31°C (Kim et al., 1996), but the biomass increased by 13–16% when the temperature increased from 25 to 28°C (Ohe et al., 2007).

To date, warming experiments have been mainly conducted at certain key rice growth stages (Cheng et al., 2009; Mohammed and Tarpley, 2009) and with few varieties (Dong et al., 2011). Few studies have encompassed the entire duration of rice growth (Nijs et al., 1996; Wan et al., 2002; Kimball and Conley, 2009) or employed different heat-resistant varieties of rice (Shah et al., 2014) simultaneously. Furthermore, most of the studies related to global warming and rice have been performed under controlled experimental conditions, for instance, in closed greenhouses (Jagadish et al., 2007) and open-topped chambers (Chiba and Terao, 2015), etc., and with temperature increases of more than 4°C, which may not accurately represent the predicted temperature increase. In addition, these facilities are convenient for investigating crop responses of a single plants or on a community scale but not on a crop system scale *in situ* (Aronson et al., 2009). As a result, the effects of warming on rice growth in previous studies might be over-estimated and inaccurate. Therefore, a field trial that encompasses the entire growth period of crop *in situ* condition is necessary to determine the actual response of the crop to the predicted warming.

China occupies a unique position among the rice-producing countries (Peng et al., 2009), and the Yangtze River Valley (YRV) is of tremendous importance to the food security because the YRV accounts for 70% of the total rice-growing area in China (Huang et al., 1998). The YRV has experienced severe rice yield losses in recent decades, particularly because of increased mid-season temperature; however, the temperature rarely exceeds 35°C (Matsui, 2009), which suggests that the critical maximum temperature range for this region is much lower for yield losses than in several other areas of the world such as Australia (Tian et al., 2010), where occasional temperatures as high as 40°C may not affect the crop production (Matsui et al., 2007). In the central and southern regions of China in the main rice-growing area of the YRV, four rice cultivars, i.e., Huanghuazhan (HHZ), Shanyou63 (SY63), Yangliangyou6 (YLY6) and Liangyoupeijiu (LYPJ), are planted widely. HHZ is a typical conventional rice variety. SY63, a three-line hybrid rice, is famous because it was the most widely planted rice cultivar in the past (Xie, 1997). YLY6 and LYPJ are both two-line hybrid rice varieties whose planting area is currently the largest in china. Interestingly, all of these rice varieties are of the indica ecotype and play an important role in the rice production in the central and southern regions of China. It has been demonstrated that a temperature rise of 2°C will result in greater losses in rice productivity and qualitative attributes than previous projected simulations for the indica and japonica ecotypes (Shah et al., 2014). However, little research has been done to compare the differences with respect to heat resistance under field conditions between these widely cultivated varieties in this area. In addition, the previous experiments were focused on the qualitative effects of elevated temperature on rice productivity, but interannual differences in the performances of rice under global warming conditions and the causes leading this phenomenon have been ignored. As an example, a heat event in 2003 along the Yangtze River in China resulted in daytime temperatures above 38°C and lasted more than 20 days, damaging an estimated 3 million hectares of rice and resulting in a loss of ~5.18 million tons of paddy rice loss (Li et al., 2004). Similar losses were recorded in the same region as a result of heat events during the 2006 and 2007 growing seasons (Zou et al., 2009). To study the response of plants to global warming on an ecosystem scale, a highly effective heating system to provide a free air temperature increase (FATI) with a far infrared heater has been extensively used (Wan et al., 2002; Kimball et al., 2008; Kimball and Conley, 2009; Dong et al., 2011). A FATI heating system provides an enormous opportunity to accurately determine the response of rice growth to warming *in situ*, but deficiencies in simulated air warming still exist due to its unstable efficiency caused by a variable weed speed. A heating apparatus known as an “open-top hot-blast system” is composed of a blower, a heater and a pipe. The warm air generated by the heater is pushed by the blower through the pipe and exists the pipe through the small holes into the plots surrounded by sunshine plates. The warm air diffuses from the bottom to up. It can nullify the effects of the outside wind flow much more effectively than the FATI. Because of these factors, we conducted a two-year field experiment with three elevated-temperature treatments via the open-top hot-blast system was conducted in the central and southern regions of China in 2015 and 2016. Our objectives were to investigate the interannual differences in rice performances under elevated temperature (by ~2°C) and the response of four widely planted indica rice varieties to elevated temperature with respect to grain yield as well as the causes of those responses under field conditions.

## Materials and methods

### Crop management

Studies were conducted at the Huazhong Agricultural University experimental field in China during the 2015 and 2016 rice growing seasons. Four widely planted rice (*Oryza sativa L*.) genotypes of the indica ecotype, i.e., Huanghuazhan (HHZ), Shanyou63 (SY63), Yangliangyou6 (YLY6) and Liangyoupeijiu (LYPJ), were used for the two-year trial. Standard practices for field preparation and crop management appropriate for the experimental site were followed. In both years, germinated certified seed for all four cultivars was sown on 14th May for the nursery rearing. One month after sowing, three seedlings per hill were transplanted with a hill spacing of 16.7 cm × 20.0 cm in 2015 and 2016. The plants were cultivated in a paddy for the entire rice growth duration. The rates of N, P, K fertilizers in each plot were 180 kg N ha^−1^, 40 kg P (P_2_O_5_) ha^−1^, 100 kg K (K_2_O) ha^−1^, respectively. All of the P and half of the K fertilizers were applied as a basal dressing, while the remaining K was applied at the panicle initiation stage. In a similar fashion, a three-way split method was used for the nitrogen application in both experiments, which was applied in a 4:3:3 ratio at the time of transplanting, mid-tillering and panicle initiation stages, respectively. All the weeds were manually removed whenever they were found, and various pesticide sprays were used for pests control.

### Experimental design and temperature treatments

**Figure 1:**
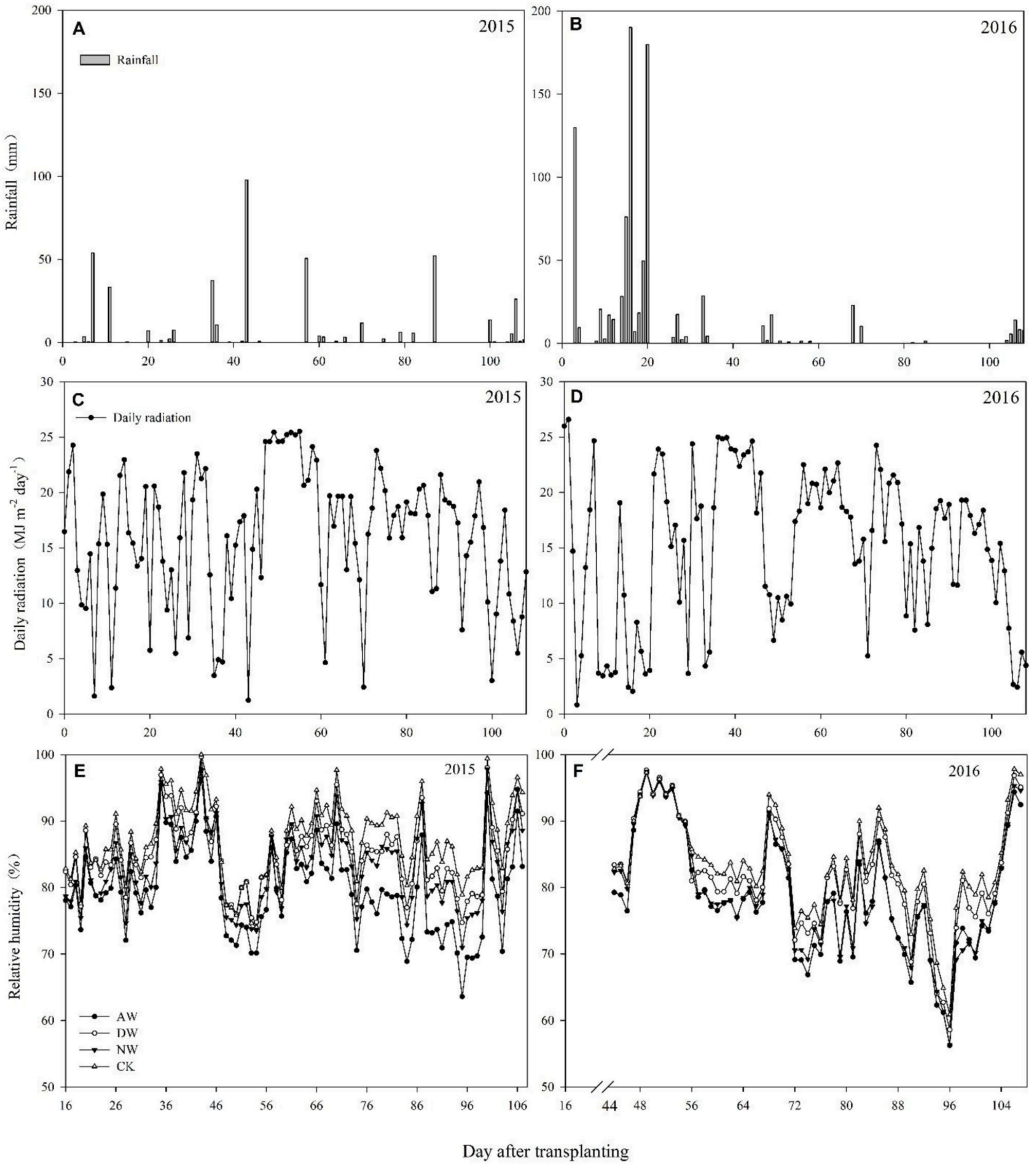
Daily rainfall, daily radiation and relative humidity in the middle rice growing season of 2015 **(A,C,E)** and 2016 **(B,D,F)**.

Studies were carried out according to split plot arrangements According to our design, elevated temperature treatments were allocated to the main-plots having an area of 25 m^2^ (5 × 5 m). Each main-plot was divided into four sub-plots equally (Supplementary Figure 3). Whereas, varieties were assigned randomly to the subplots. The field temperature was augmented by an open-top hot-blast system (Supplementary Figure 1). To maintain constant elevated temperatures inside the plot that were different from the rest of the field, sunshine plates with a constant height of 110 cm were used to surround the plots from the initiation of the elevated-temperature treatments until harvesting time. A front covering was attached to 450 cm-long PVC pipes with an inner diameter of 11 cm. Two such heating systems (blower + heater + pipe) were positioned at the two opposite sides across the length of the main-plots (Supplementary Figure 2). A local company was employed to design the heater size and shape to allow the easy connection of the heater to the blower as well as heater adjustment inside the iron cover of the system. Each individual heater installed had a power of 1.5 kW. Small holes of ~0.8–1.0 cm diameter were made at several places on the pipes to allow the warm air generated by the heater and pushed by the blower to exit the pipe and increase the temperature of the plots. Four different elevated temperatures were assigned to the plots, i.e., the control (ambient temperature), NW (night-time warming), DW (daytime warming) and the AW (all-day warming) treatments. The heating systems was operated all day and night for the AW treatment. The heating system was started at 7:00 AM and turn off at 7:00 PM for the DW treatment and vice versa for the NW treatment. No heating system was installed for the control plots, but they were surrounded with sunshine plates in a similar manner to the other plots in order to provide better homogeneity of factors excluding temperature. The elevated-temperature treatments were employed after transplanting, from 25/06/2015 to 29/09/2015 and from 29/07/2016 to 08/10/2016, to assess the response of the rice to the elevated temperature for the entire duration of growth. The start time of the elevated-temperature treatments was delayed because of the continual rainfall after transplanting in 2016. A HOBO Micro Station Onset Computer Corporation Data Logger (H21–002) with different sensors (12-Bit Temp Smart Sensor S-TMB-017, which can detect temperatures from −40 to 100°C, Onset Computer Corporation, Massachusetts, USA) was fixed in the field to recording the temperature and relative humidity data. Sensors connecting a coupling were attached to bamboo poles at the height of the rice canopy by regulating the coupling as required. All the sensors were sheltered from rain, sunlight and other environmental conditions by utilizing solar radiation shields to achieve more precise data,. The data for daily rainfall and solar radiation during the rice growing season were collected at a meteorological station (CR800, Campbell Scientific Inc., Logan, Utah, USA) near the fields and are shown in Figure 1.

### Pollen viability

Pollen viability was determined using the I_2_-KI staining method (Wang et al., 2006). Pre-flowering spikelets were sampled and stored in a −20°C refrigerator. Twelve anthers were sampled randomly and then removed from the spikelets. The pollen was placed on slides with 1% I_2_-KI solution and cut into pieces using forceps to make the pollen grains spill out. The pollen viability was assessed in an inverted fluorescence microscope based on staining and morphology. Pollen grains that were round in shape and stained black were considered to be viable.

### Pollen grain retention and germination on the stigma

After flowering, 20 florets from each temperature treatment of the four genotypes were sampled and the number of pollen grains deposited on the stigmas was counted. The spikelets were washed in de-ionized water before dissecting under an inverted fluorescence microscope. The isolated stigmas were cleaned in 8N NaOH for 3–5 h at room temperature and stained with aniline blue dissolved in 0.1 M K_2_HPO_4_ for 5–10 min. The number of pollen grains adhering to stigma and the germinated pollen grains (i.e., those that had a pollen tube emerging from the pollen coat) on the stigma were recorded. The examination was done at 40 × total magnification with an inverted fluorescence microscope. Images were taken with a DP70 digital camera attached to a Carl Zeiss micro-scope (Ti-E, Nikon Instech Co. LTD, Minato-ku, Tokyo, Japan).

### Dry matter

The heading date, defined as the date when more than 50% of all panicles had emerged from the flag leaf sheath, was determined. The dry weight of the aboveground parts was measured by sampling the plants at the heading stages for eight hills per plant in each plot. The plants were separated into straw and panicles. The straw was further divided into leaves and stems and then oven dried at 80°C to constant weight, after which the dry weight was determined.

### Yield and its components

Ten hills in each subplot from the central rows were collected at maturity in both years to measure the grain yield, the aboveground total biomass, the HI and the yield components. To measure the panicle number per m^2^, all of the panicles collected from each hill were counted. The plants were separated into straw and panicles. The straw was further divided into leaves and stems and then oven dried at 80°C to constant weight, after which the dry weights was determined. The panicles were hand threshed, and all of the filled spikelets were separated from the unfilled spikelets using a seed blower. The number of spikelets was measured from the three sub-samples. Each sub samples had 30-g filled spikelets and 5-g unfilled spikelets. The spikelets per panicle, spikelets per m^2^ and grain-filling percentage (100 × filled spikelet number/total spikelet number) were also measured. Moreover, all of the panicles were harvested randomly from 50 hills in the central rows at maturity stage in both years for the grain yield determination. All of the panicles were subsequently hand threshed manually, and the grains were air dried for 3 days. The moisture content of grains was tested with a digital moisture meter by randomly picking three sub-samples and extrapolated for the entire sample. The grain yield was then adjusted to a moisture content of 0.14 g H_2_O g^−1^ fresh weight.

### Statistical analysis

All treatments were replicated four times in both years. The data were statistically analyzed by an analysis of variance (ANOVA; SAS statistical analysis system, version 9.2; SAS Institute, Cary, NC, USA) to determine the significance of the various treatments. The means were separated using the least significance difference (LSD) at an alpha level of 0.05. All figures were drawn with Sigmaplot software (version 12.5) (Systat Software, Inc., San Jose, California, USA)

## Results

### Temperature increase

The device developed in the present study for heating the plots could raise the air temperature in the canopy by ~2°C higher than that of the control. The data shown for each treatment are the averaged of four independent plots (Figure 1). The air temperature in 2016 was higher than that in 2015 during the rice-growthing period. During the booting stage, the night-time temperature of the NW treatment, the daytime temperature of the DW treatment, the diel temperature of the AW treatment were ~28.1, 32.2, and 29.4°C in 2015 and 28.2, 32.1, and 30.1°C in 2016, respectively, values that were close to the heat stress threshold for this stage. During the anthesis stage, the night-time temperature of the NW treatment, the daytime temperature of the DW treatment, and the night-time and the daytime temperature of the AW treatment were 27.0, 30.2, 26.9, and 30.2°C, respectively. In contrast, the values for 2016 were ~30.1, 34.3, 29.9, and 34.4°C, respectively. However, the temperature during the filling stage in 2015 and 2016 were similar. Specifically, the night-time temperature of the NW treatment, the daytime temperature of the DW treatment, and the night-time and the daytime temperature of the AW treatment were 24.1, 28.5, 24.9, and 29.3°C in 2015 and 24.4, 28.3, 23.8, and 28.5°C in 2016, respectively.

### Dry matter accumulation

No significant differences in dry matter accumulation were noted at the pre-anthesis and post-anthesis stages for any elevated-temperature treatment in both 2015 and 2016 (Table 1). A comparison of 2015 and 2016 with respect to dry matter accumulation in the pre and post-anthesis stages and for the entire duration revealed a significant difference between the 2 years. The dry matter accumulation of the entire duration in 2015 was much higher than in 2016 because of greater dry matter accumulation in the pre-anthesis stage in 2015. However, dry matter accumulation in 2016 at the post-anthesis stage was significantly higher than in 2015.

**Table 1:** Dry matter accumulation at the pre-anthesis and post-anthesis stages in different elevated-temperature treatments and the effect of elevated temperature treatments on HI in 2015 and 2016.

**Variety**	**Treatment**	**2015**				**2016**			
		**Dry matter accumulated(g/m**^**2**^**)**			**Harvest Index (HI)**	**Dry matter accumulated(g/m**^**2**^**)**			**Harvest Index (HI)**
		**Pre-anthesis**	**Post-anthesis**	**Whole duration**		**Pre-anthesis**	**Post-anthesis**	**Whole duration**	
HHZ	CK	973.9 ± 46.2 a	558.3 ± 114.4 a	1,532.2 ± 78.8 a	54.1 ± 0.5 a	693.1 ± 29.2 a	639.4 ± 110.6 a	1,332.5 ± 136.8 a	43.5 ± 1.6 a
	NW	935.3 ± 45.8 a	414.6 ± 43.7 a	1,349.9 ± 80.0 a	51.4 ± 1.1 a	738.4 ± 52.5 a	541.5 ± 95.0 a	1,279.9 ± 53.8 a	37.0 ± 2.9 ab
	DW	988.9 ± 26.3 a	426.1 ± 20.7 a	1,415.1 ± 45.0 a	52.6 ± 0.4 a	655.5 ± 19.6 a	367.6 ± 46.7 a	1,023.0 ± 35.7 b	32.1 ± 2.1 b
	AW	940.3 ± 11.5 a	445.4 ± 85.8 a	1,385.6 ± 90.3 a	41.0 ± 3.8 b	731.2 ± 67.8 a	537.3 ± 135.2 a	1,268.5 ± 92.6 a	15.1 ± 5.4 c
SY63	CK	1,192.9 ± 58.2 a	533.8 ± 36.2 a	1,726.7 ± 83.8 a	54.2 ± 0.8 a	921.7 ± 25.4 a	766.2 ± 130.4 a	1,687.9 ± 109.1 a	43.4 ± 0.6 a
	NW	1,328.2 ± 51.7 a	569.5 ± 33.4 a	1,897.7 ± 49.6 a	51.6 ± 1.5 ab	934.2 ± 57.9 a	504.7 ± 18.2 a	1,438.9 ± 52.3 a	31.3 ± 4.9 ab
	DW	1,276.0 ± 57.1 a	500.3 ± 95.5 a	1,776.3 ± 47.2 a	54.5 ± 0.5 a	971.2 ± 76.2 a	439.8 ± 64.7 a	1,411.0 ± 35.6 a	36.9 ± 4.8 ab
	AW	1,367.8 ± 28.2 a	513.0 ± 199.4 a	1,840.1 ± 172.5 a	46.5 ± 4.6 b	999.7 ± 58.3 a	553.0 ± 138.8 a	1,552.7 ± 128.9 a	26.2 ± 3.9 b
YLY6	CK	1,228.2 ± 77.3 a	727.1 ± 91.3 a	1,955.3 ± 30.6 a	56.5 ± 0.4 a	1,003.5 ± 34.5 a	692.4 ± 47.8 a	1,695.9 ± 33.6 a	52.5 ± 0.8 a
	NW	1,327.5 ± 64.4 a	445.7 ± 93.6 a	1,773.2 ± 81.9 b	55.0 ± 0.8 a	1,093.2 ± 82.6 a	608.2 ± 52.7 a	1,701.3 ± 75.7 a	44.0 ± 3.1 ab
	DW	1,187.9 ± 27.5 ab	568.9 ± 50.8 a	1,756.8 ± 51.9 b	53.6 ± 1.1 a	1,011.1 ± 69.2 a	489.1 ± 160.1 a	1,500.2 ± 108.3 a	49.0 ± 1.7 a
	AW	1,105.8 ± 41.1 b	573.2 ± 54.1 a	1,679.0 ± 33.2 b	47.5 ± 2.4 b	1,056.8 ± 46.1 a	471.3 ± 57.2 a	1,528.1 ± 23.0 a	27.4 ± 10.8 b
LYPJ	CK	1,267.4 ± 48.0 ab	392.9 ± 80.5 a	1,660.4 ± 63.8 a	54.9 ± 0.5 a	1,129.4 ± 60.3 a	483.3 ± 52.4 a	1,612.7 ± 54.8 a	40.9 ± 0.9 a
	NW	1,231.7 ± 17.2 ab	538.6 ± 43.8 a	1,770.3 ± 52.9 a	53.6 ± 1.2 a	1,121.2 ± 48.0 a	478.3 ± 57.9 a	1,599.5 ± 31.9 a	18.9 ± 3.6 b
	DW	1,327.2 ± 61.7 a	514.1 ± 116.2 a	1,841.3 ± 72.3 a	48.1 ± 1.3 ab	983.5 ± 45.6 b	474.3 ± 36.0 a	1,457.8 ± 76.7 a	24.1 ± 5.0 b
	AW	1,199.5 ± 44.5 b	494.5 ± 61.0 a	1,694.0 ± 35.9 a	39.0 ± 8.4 b	993.9 ± 49.6 b	640.1 ± 92.8 a	1,634.0 ± 57.0 a	15.6 ± 1.4 b

*In the column for each variety, means followed by different letters are significantly different at the 0.05 probability level according to the least significant difference (LSD) test. Abbreviations: HHZ, Huanghuazhan; SY63, Shanyou63; YLY6, Yangliangyou6; LYPJ, Liangyoupeijiu. CK, ambient control; NW, night-time warming; DW, daytime warming; AW, and all-day warming treatments. Eight hills were used to measure the biomass in the pre-anthesis and ten hills were used to measure the biomass and HI in the maturity stage*.

### Harvest index

The harvest index (HI) of all varieties was significantly (*P* < 0.05) affected by the elevated temperature treatments (Table 1). The AW treatments had a more pronounced effect on the HI of all varieties than the DW and NW treatments and the control in both 2015 and 2016. The DW and NW treatments also tended to reduce the HI. The HI was respectively 3.64 and 4.92% lower in the DW and NW treatments than in the control on average for the four genotypes in 2015. Similar to 2015, all elevated-temperature treatments significantly reduced the HI of all four varieties in 2016. Considering the genotypic variation, the data revealed that YLY6 had the highest mean of all elevated-temperature treatments in 2015 (53.2%) and 2016 (43.2%), and LYPJ had the lowest HI both in 2015 (48.9%) and 2016 (24.9%). Moreover, the HI in the control in 2016 was as low as in the AW treatment in 2015.

### Grain yield

The AW treatments significantly affected the grain yield of all the genotypes compared to the control treatment in both 2015 and 2016, except for that of SY63 in 2015. The reduction caused by the AW treatments was greater for all cultivars than the NW or DW treatments in both years (Table 2). The average yield of all varieties was decreased by 4.37, 5.02, and 22.7% in the NW, DW and the AW treatments, respectively, in 2015, while a severe reduction of 30.76, 31.06, and 61.93% occurred in the NW, DW and AW treatments in 2016, respectively. The effects of the DW and NW treatments on the yield were not significant in 2015, but the NW treatment significantly reduced the yield of SY63, YLY6 and LYPJ, and the DW treatment significantly reduced the yield of HHZ and LYPJ in 2016. Considering the genotypic variation, the data shown that the grain yield reduction in SY63 was the lowest of all four varieties in the NW, DW, and AW treatments in both years; however, the reduction in LYPJ was the highest in the elevated-temperature treatments of all varieties. The data in Table 2 clearly show that the yield of the first year was significantly higher than in the second year. In 2015, the overall yields in the control and the AW treatment were more than 10 t/ha and 8 t/ha, respectively, while in 2016, the yields of the control and the AW treatment were <8 t/ha and 3 t/ha, respectively, when averaged for all genotypes. The mean yield in the control in 2016 was lower than the mean yield in the AW treatment in 2015.

**Table 2:** Effects of different elevated temperature treatments on grain yield (t/ha) of the genotypes in field experiments in 2015 and 2016.

**Year**	**Treatment**	**Variety**				**Diff(%)**				**Mean**
		**HHZ**	**SY63**	**YLY6**	**LYPJ**	**HHZ**	**SY63**	**YLY6**	**LYPJ**	
2015	CK	8.76 ± 0.32a	10.75 ± 0.55a	10.91 ± 0.27a	11.32 ± 0.18a	–	–	–	–	–
	NW	7.90 ± 0.58ab	10.35 ± 0.72a	11.26 ± 0.42a	10.51 ± 0.40a	9.82	3.73	−3.18	7.12	4.4
	DW	8.68 ± 0.30a	10.20 ± 0.24a	11.11 ± 0.38a	9.52 ± 0.24ab	0.91	5.13	−1.84	15.87	5.0
	AW	6.47 ± 0.78b	8.71 ± 1.22a	9.76 ± 0.43b	7.34 ± 1.57b	26.14	19.02	10.51	35.13	22.7
2016	CK	6.01 ± 0.22a	7.54 ± 0.09a	9.90 ± 0.33a	7.77 ± 0.11a	–	–	–	–	–
	NW	4.94 ± 0.50ab	5.40 ± 0.62b	7.72 ± 0.50b	3.51 ± 0.41b	17.80	28.38	22.02	54.83	30.8
	DW	4.43 ± 0.21b	5.60 ± 0.43ab	7.91 ± 0.18ab	3.72 ± 0.51b	26.29	25.73	20.10	52.12	31.1
	AW	2.05 ± 0.33c	4.30 ± 0.52b	3.86 ± 1.11c	1.72 ± 0.21c	65.89	42.97	61.01	77.86	61.9
Year		^***^	^***^	^***^	^***^					
Temperature		^***^	^**^	^***^	^***^					
Year*Temperature		ns	ns	^**^	ns					

*In the column for each variety, means followed by different letters are significantly different at the 0.05 probability level according to the least significant difference (LSD) test*.

“*, **, and ***” *denote significance at the 0.05, 0.01, and 0.001 probability levels based on analysis of variance, respectively. “ns” denotes non-significant based on analysis of variance. Abbreviations: HHZ, Huanghuazhan; SY63, Shanyou63; YLY6, Yangliangyou6; LYPJ, Liangyoupeijiu. CK, ambient control; NW, night-time warming; DW, daytime warming; AW, and all-day warming treatments. Fifty hills were handled to measure the actual yield in the maturity stage*.

### Yield components

There were significant reductions in the number of spikelets per panicle of HHZ and YLY6 in the AW treatment in 2015 (Table 3). The indica variety SY63 produced fewer spikelets per panicle in the NW (24.78%), DW (20%) and the AW treatments (20%) than in the control in 2016 (Table 5). For the panicle number per m^2^, no significant variations were observed in any variety in 2015; however, in 2016, the maximum number of panicles per m^2^ for HHZ was found in the AW treatment and the minimum was observed in the NW treatment. The effect of the DW treatment on the panicle number of YLY6 and LYPJ was greater than in the control.

**Table 3:** Effects of different elevated temperature treatments on the yield components of the genotypes in the field experiments in 2015.

**Variety**	**Treatment**	**Spikelets per panicle**	**Panicles m^−2^**	**Spikelets m^−2^ (10^3^)**	**Grain filling percentage (%)**	**Grain weight (mg)**
HHZ	CK	182 ± 3.93a	259 ± 13.08a	47.0 ± 2.67a	87.6 ± 1.57a	20.2 ± 0.16a
	NW	176 ± 8.20ab	249 ± 7.02a	43.8 ± 2.55a	81.8 ± 1.53ab	19.3 ± 0.11b
	DW	187 ± 3.13a	243 ± 8.76a	45.3 ± 1.06a	83.7 ± 1.97ab	19.6 ± 0.13b
	AW	151 ± 13.77b	264 ± 3.25a	40.0 ± 3.93a	77.7 ± 4.53b	18.4 ± 0.21c
SY63	CK	179 ± 10.05a	211 ± 3.96a	37.7 ± 1.69a	90.5 ± 1.40a	27.4 ± 0.15a
	NW	187 ± 6.75a	218 ± 7.00a	40.7 ± 1.16a	90.8 ± 1.22a	26.5 ± 0.26ab
	DW	197 ± 7.53a	203 ± 1.82a	40.0 ± 1.56a	90.5 ± 1.31a	26.9 ± 0.13ab
	AW	184 ± 12.80a	218 ± 3.30a	40.1 ± 3.28a	82.2 ± 7.75a	25.9 ± 0.57b
YLY6	CK	197 ± 3.49a	226 ± 1.93a	44.6 ± 1.08a	87.4 ± 1.53a	28.4 ± 0.29a
	NW	192 ± 7.77a	216 ± 5.99a	41.5 ± 2.35ab	87.8 ± 1.77a	26.7 ± 0.34b
	DW	199 ± 4.09a	226 ± 7.58a	45.1 ± 2.19a	79.2 ± 2.26ab	26.5 ± 0.51b
	AW	177 ± 1.43b	226 ± 5.11a	40.0 ± 0.87b	76.4 ± 5.65b	26.3 ± 0.42b
LYPJ	CK	219 ± 7.32a	223 ± 3.77a	48.9 ± 2.35a	75.4 ± 2.73ab	24.8 ± 0.24a
	NW	203 ± 5.19a	236 ± 4.95a	47.8 ± 1.13a	82.3 ± 1.51a	24.1 ± 0.20ab
	DW	207 ± 7.02a	243 ± 4.95a	50.3 ± 1.13a	73.6 ± 4.34ab	24.0 ± 0.53ab
	AW	218 ± 8.26a	240 ± 12.76a	52.0 ± 1.31a	56.6 ± 13.26b	22.8 ± 0.37b

*In the column for each variety, means followed by different letters are significantly different at the 0.05 probability level according to the least significant difference (LSD) test. Abbreviations: HHZ, Huanghuazhan; SY63, Shanyou63; YLY6, Yangliangyou6; LYPJ, Liangyoupeijiu. CK, ambient control; NW, night-time warming; DW, daytime warming; AW, and all-day warming treatments. Ten hills were used to measure the yield components in the maturity stage*.

No variation in the spikelets number per m^2^ was observed except for the significant reduction in YLY6 in the AW treatment compared to the control in 2015. However, the data for 2016 demonstrated that the elevated-temperature treatments significantly reduced the spikelet number per m^2^. The DW treatment-associated reduction was greater than in the NW and the AW treatments for the spikelet number per m^2^ for all varieties. The reduction in the spikelet number per m^2^ of HHZ, SY63, YLY6, and LYPJ in the DW treatment was 18.59, 25.32, 9.07, and 10.23%, respectively. The spikelet number per m^2^ of SY63 in the NW treatment and YLY6 in the AW treatment were lower than in the control.

The filling rate of all varieties was significantly lower in response to the different elevated-temperature treatments in 2015. Of all treatments, the AW treatment had the greatest effect on the filling rate of all four varieties. The AW treatment significantly reduced the filling rate in all varieties except for the SY63. The reduction of the filling rate of HHZ, SY63, YLY6, and LYPJ in the AW treatment was 11.38, 9.24, 12.60, and 24.97%, respectively, in 2015; however, it was more severe in 2016 for HHZ (64.32%), SY63 (26.01%), YLY6 (46.31%), and LYPJ (59.82%). The reduction of the filling rate of HHZ, SY63, YLY6, and LYPJ in the NW and DW treatments was 8.35, 12.48, 11.79, and 50.37% and 29.21, 2.11, 7.14, and 42.13%, respectively. The reduction of the filling rate of HHZ in the DW treatment and LYPJ in both the NW and DW treatments was significant. A comparison of the 2 years revealed that the filling rates were considerably lower in 2016 for all elevated-temperature treatments than in 2015.

The grain weight was reduced in all elevated-temperature treatments compared to the control, and the reduction in the AW treatment was the greatest in 2015. The NW and DW treatments reduced the grain weight significantly for HHZ and YLY6 compared to the control. In 2016, the grain weight of HHZ and SY63 was significantly reduced in the AW treatment compared to the control; however, no significant differences were noted for the YLY6 and LYPJ in any of the elevated-temperature treatments compared to the control. There was a significant decrease in the grain weight in 2016 compared to that in 2015. All the grain weights of the varieties were much lower in 2016 than in 2015; even the grain weights of the HHZ and LYPJ cultivars in the control in 2016 were slightly less than in the AW treatment in 2015.

### Pollen viability

**Figure 2:**
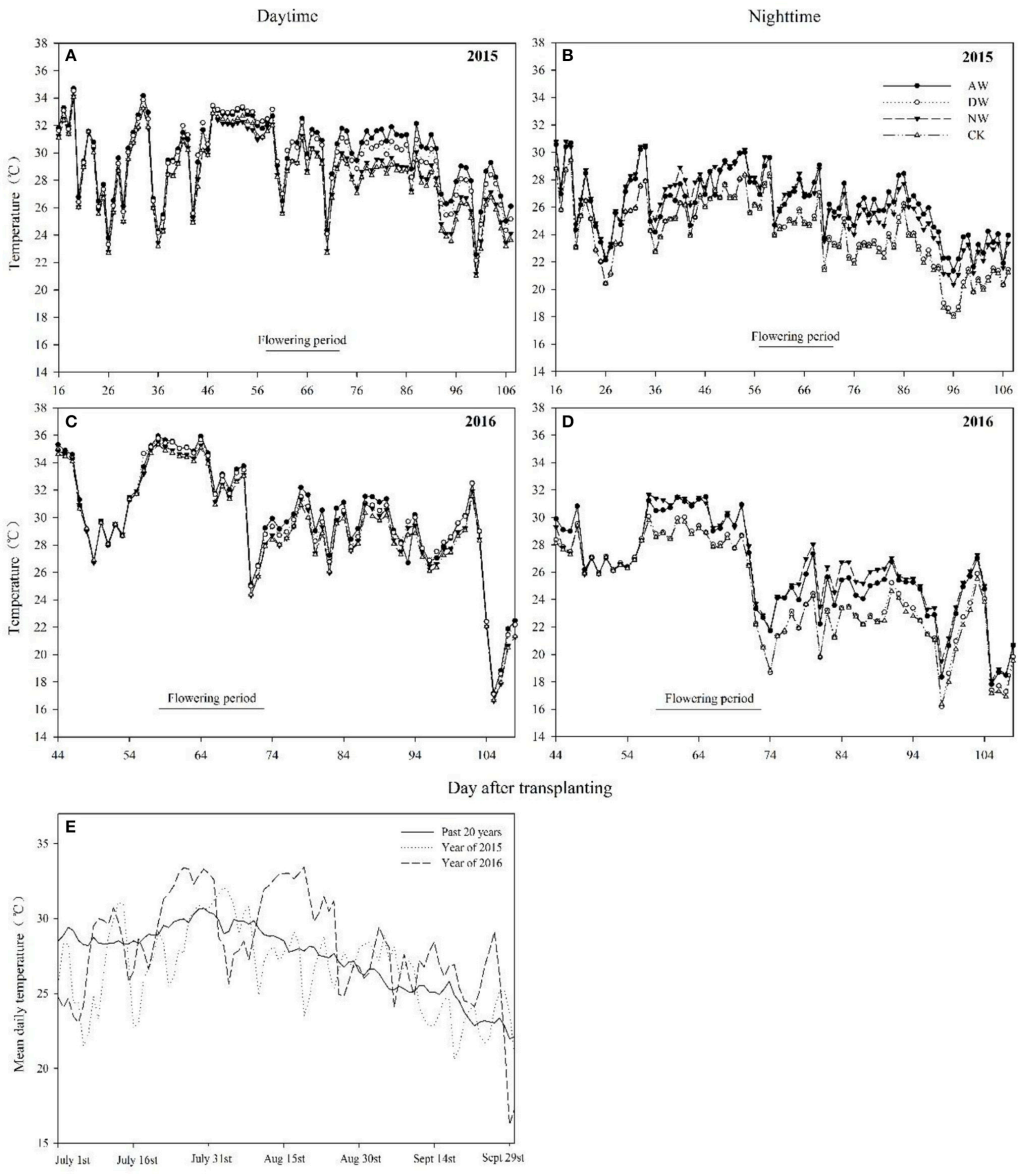
Mean day time and night time temperatures in different elevated-temperature treatments during two experimental years **(A–D)**. Day mean temperatures for the past 20 years from 1994 to 2014 as well as the mean day temperature of air in 2015 and 2016 **(E)**. CK, ambient control; NW, night-time warming; DW, daytime warming; AW, and all-day warming treatments. The horizontal axis represents the days after transplanting.

The AW treatment significantly reduced the pollen viability compared to the control for all the genotypes in both 2015 and 2016 (Figure 2). The pollen viability of HHZ was reduced significantly by both the NW and DW treatments compared to the control, and no significant differences in the pollen viability for SY63, YLY6, or LYPJ were observed in 2015. In 2016, the pollen viability of HHZ and LYPJ in the DW treatment was significantly less than in the control, while the NW treatment only significantly impacted the pollen viability of SY63. The percentage of non-viable pollen grains in 2015 was not more than 20%, which was considerably lower than that in 2016 (46%). Of all genotypes, LYPJ was the most sensitive to elevated temperature with respect to pollen viability.

### Pollen grain retention and germination on the stigma

**Figure 3:**
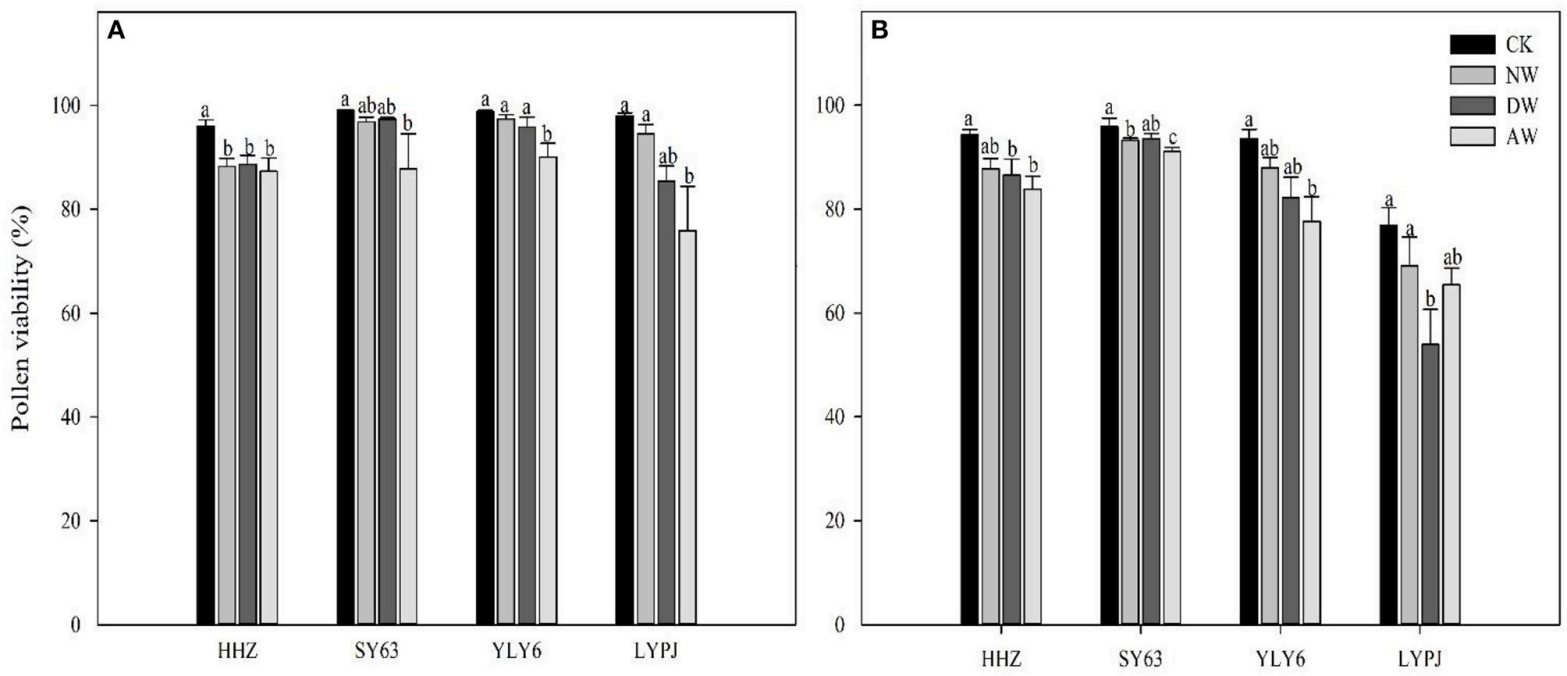
Effects of different elevated temperature treatments on the pollen viability of the varieties in 2015 **(A)** and 2016 **(B)**. Abbreviations: HHZ, Huanghuazhan; SY63, Shanyou63; YLY6, Yangliangyou6; LYPJ, Liangyoupeijiu. CK, ambient control; NW, night-time warming; DW, daytime warming; AW, and all-day warming treatments. Means followed by different letters are significantly different at a probability level of 0.05 according to the least significant difference (LSD) test. Error bars above denote the standard error (SE) of the replicates (*n* = 4).

The number of pollen grains adhering to the stigma was drastically reduced by the elevated-temperature treatments in both 2015 and 2016. The data in the AW treatment showed significantly reduced pollen numbers adhering to the stigma compared to the control in 2015 for all genotypes. In 2015, no significant differences were observed in the NW and DW treatments compared to the control, except for HHZ and SY63 in the NW treatment. However, in 2016, all the elevated-temperature treatments significantly reduced the number of pollen grains adhering to stigma for HHZ, SY63, and LYPJ. In contrast, only the AW treatment significantly reduced the number of pollen grains adhering to the stigma for YLY6. LYPJ was the most severely affected of all varieties by elevated temperature with respect to the number of pollen grains adhering to the stigma in both 2015 (Figure 3A) and 2016 (Figure 3B).

**Figure 4:**
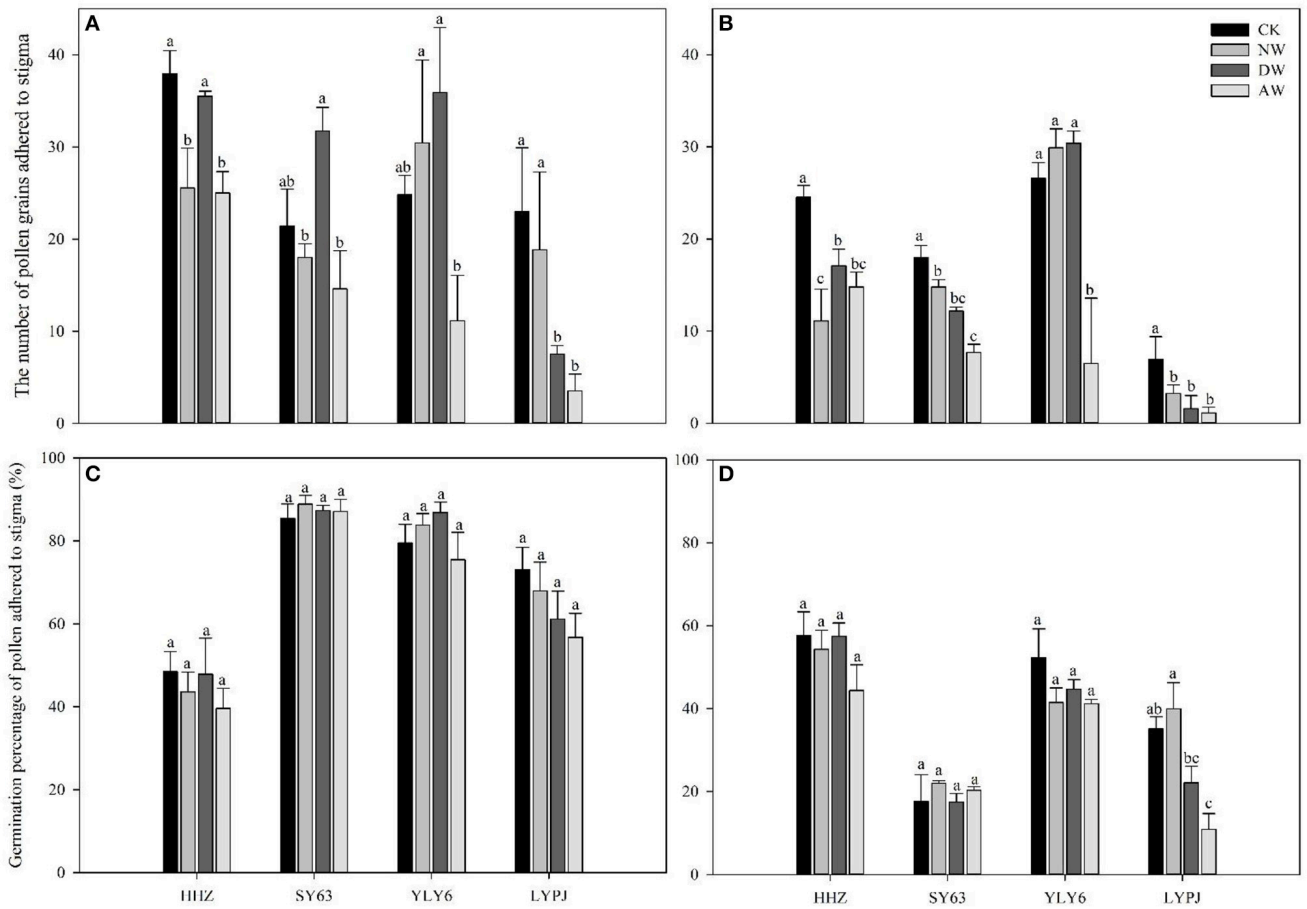
Effects of different elevated temperature treatments on the number of pollen grains adhering to the stigma in 2015 **(A)** and 2016 **(B)** and the number of germinated pollen grains on the stigma of the varieties in 2015 **(C)** and 2016 **(D)**. Abbreviations: HHZ, Huanghuazhan; SY63, Shanyou63; YLY6, Yangliangyou6; LYPJ, Liangyoupeijiu. CK, ambient control; NW, night-time warming; DW, daytime warming; AW, and all-day warming treatments. Means followed by different letters are significantly different at a probability level of 0.05 according to the least significant difference (LSD) test. Error bars above denote the standard error (SE) of the replicates (*n* = 4).

No significant differences in the germination percentage of pollen grains on the stigma were observed in the AW, NW, DW, and control treatments for any genotypes in 2015 or 2016. However, the germination percentage of pollen grains was obviously higher in 2015 than 2016. Of the various cultivars, LYPJ was the most sensitive with respect to the germination percentage of pollen grains, while YLY6 was most tolerant (Figures 4C,D).

## Discussion

Our results showed that a higher temperature significantly reduced the rice yield under field conditions (Table 2). Similar results were obtained in previous studies (Ohe et al., 2007; Shah et al., 2014; Chaturvedi et al., 2017). However, a close relationship between the range of yield reduction and the annual temperature was apparent. In detail, the decrease in rice yield caused by elevated temperature was significantly higher in 2016 (43–78% in the AW treatment) than in 2015 (11–35% in the AW treatment). Rice grain yield is determined by four parameters: the number of panicles per plant, the number of spikelets per panicle, the grain filling rate and the total grain weight. The yield reduction in the present field experiment was mainly attributed to a decrease in the 1,000-grain weight in 2015, while the filling rate was also impacted by the higher temperatures (Table 3). Nevertheless, the percentage of the reduction in grain weight in the elevated-temperature treatments compared to the control shown that the reduction caused by the elevated temperature treatments was obviously higher in 2015 than in 2016. The grain weight of the rice in the control in 2016 was already impacted by an unfavorable environment, so there is no doubt that elevated temperature caused the significant decrease in the filling rate and the 1,000-grain weight in 2016 (Table 4), which indicated considerable heat stress on both the spikelet fertilization and grain filling. The main reason for the diverse performances in yield and yield components between 2015 and 2016 was the considerable differences in the air temperature between the 2 years. The variation in the air temperature during the entire duration of rice growth in 2015 was similar to the variation trend of the air temperature during the 20-years period from 1996 to 2016. However, the mean temperature in 2016 was obviously higher than that in the previous 20 years (Figure 2). According to our findings, the reduction recorded in the grain weight of all four rice cultivars can be ascribed to a higher temperature, particularly during the ripening period (Morita, 2008; Fahad et al., 2016a). Although the rice grain carbohydrates are mainly derived from the photosynthetic products during the post-anthesis phase, the dry matter accumulated during pre-anthesis also plays a role in the accumulation of rice grain carbohydrates to some degree. Generally, high temperature impacts crop biomass production and grain yield by shortening crop growth period (Porter and Gawith, 1999), stimulating crop respiration (Schlenker and Roberts, 2009) and causing heat stress on spikelet fertilization and grain filling (Lele, 2010). However, in the present study, no significant reduction of crop biomass production either pre- or post-anthesis was observed in 2015 or 2016 (Table 1), which demonstrated that the limiting factor for grain filling is not the supply of accumulated nutrients. During the onset of fertilization and the post-anthesis period, if the daytime temperatures are higher than a certain threshold, flag leaf photosynthesis decreases substantially, resulting in disturbed source-sink assimilate transport. As a result, the high temperature reduces the grain size and weight (Cooper et al., 2008; Mohammed and Tarpley, 2010) by reducing the endosperm sink strength and incomplete grain-filling events due to reduction in the grain endosperm cell size and the grain growth rate (Morita et al., 2005). Deterioration of the grain weight at high temperatures is accompanied by the altered expression of starch metabolism-related genes. The rice grain in incomplete filling 1 (GIF1) gene that encodes a cell wall invertase (CWI) required to cleave sucrose into hexoses for carbon partitioning during early grain filling has been successfully targeted to improve grain weight during cultivation (Wang et al., 2008).

**Table 4:** Effects of different elevated-temperature treatments on the yield components of the genotypes in field experiments in 2016.

**Variety**	**Treatment**	**Spikelets per panicle**	**Panicles m^−2^**	**Spikelets m^−2^ (10^3^)**	**Grain filling percentage (%)**	**Grain weight (mg)**
HHZ	CK	175 ± 2.43a	269 ± 20.06ab	47.4 ± 4.19a	69.5 ± 3.16a	17.6 ± 0.10a
	NW	176 ± 6.93a	240 ± 8.84b	42.0 ± 0.14ab	63.7 ± 2.94ab	17.6 ± 0.22ab
	DW	159 ± 8.39a	243 ± 6.70ab	38.6 ± 1.80b	49.2 ± 3.53b	17.3 ± 0.03ab
	AW	161 ± 7.20a	284 ± 21.24a	45.6 ± 3.32ab	24.8 ± 8.95c	17.1 ± 0.15b
SY63	CK	230 ± 11.70a	220 ± 5.68a	50.6 ± 3.23a	56.9 ± 2.08a	25.4 ± 0.02a
	NW	173 ± 3.78b	210 ± 6.38a	36.4 ± 1.84b	49.8 ± 7.78a	24.9 ± 0.21ab
	DW	184 ± 19.84b	206 ± 8.27a	37.8 ± 3.43b	55.7 ± 5.96a	24.7 ± 0.23ab
	AW	184 ± 15.88b	217 ± 10.38a	39.9 ± 4.14ab	42.1 ± 6.52a	24.5 ± 0.34b
YLY6	CK	189 ± 7.98a	207 ± 9.45ab	38.8 ± 0.90a	84.0 ± 1.05a	27.3 ± 0.10a
	NW	190 ± 5.34a	199 ± 10.16ab	37.7 ± 1.02ab	74.1 ± 6.03a	26.8 ± 0.18a
	DW	192 ± 7.16a	183 ± 7.73b	35.3 ± 2.57ab	78.0 ± 2.60a	26.7 ± 0.24a
	AW	162 ± 12.88a	214 ± 11.16a	34.1 ± 0.91b	45.1 ± 17.16b	26.6 ± 0.44a
LYPJ	CK	177 ± 13.44a	254 ± 19.68a	44.3 ± 1.31a	66.7 ± 2.97a	22.4 ± 0.16a
	NW	175 ± 5.52a	237 ± 6.60ab	41.3 ± 1.53ab	33.1 ± 6.50b	22.2 ± 0.14a
	DW	181 ± 5.02a	219 ± 9.97a	39.7 ± 2.03b	38.6 ± 7.07b	22.5 ± 0.23a
	AW	183 ± 3.43a	234 ± 5.08ab	42.7 ± 0.79ab	26.8 ± 2.30b	22.2 ± 0.13a

*In the column for each variety, means followed by different letters are significantly different at the 0.05 probability level according to the least significant difference (LSD) test. Abbreviations: HHZ, Huanghuazhan; SY63, Shanyou63; YLY6, Yangliangyou6; LYPJ, Liangyoupeijiu. CK, ambient control; NW, night-time warming; DW, daytime warming; AW, and all-day warming treatments. Ten hills were used to measure the yield components in the maturity stage*.

**Table 5:** The two-way ANOVA for factors: year (Y) and temperature (T).

**Source**	**HI (%)**				**Spikelets per panicle**				**Panicles m**^**−2**^				**Spikelets m**^**−2**^ **(10**^**3**^**)**				**Grain filling percentage (%)**				**Grain weight (mg)**			
	**HHZ**	**SY63**	**YLY6**	**LYPJ**	**HHZ**	**SY63**	**YLY6**	**LYPJ**	**HHZ**	**SY63**	**YLY6**	**LYPJ**	**HHZ**	**SY63**	**YLY6**	**LYPJ**	**HHZ**	**SY63**	**YLY6**	**LYPJ**	**HHZ**	**SY63**	**YLY6**	**LYPJ**
Y	^***^	^***^	^**^	^***^	ns	ns	ns	^***^	ns	ns	^***^	ns	ns	ns	^***^	^***^	^***^	^***^	^*^	^***^	^***^	^***^	ns	^***^
T	^***^	^**^	^**^	^***^	^*^	ns	^**^	ns	ns	ns	ns	ns	ns	ns	ns	ns	^***^	ns	^**^	^**^	^***^	^**^	^**^	^**^
Y × T	ns	ns	ns	ns	ns	^*^	ns	ns	ns	ns	ns	ns	ns	ns	ns	ns	^***^	ns	ns	^*^	^**^	ns	ns	^*^

“*, ** and ***” *denote significance at the 0.05, 0.01, and 0.001 probability levels based on an analysis of variance, respectively. “ns” denotes non-significant based on an analysis of variance. Abbreviations: HHZ, Huanghuazhan; SY63, Shanyou63; YLY6, Yangliangyou6; LYPJ, Liangyoupeijiu. CK, ambient control; NW, night-time warming; DW, daytime warming; AW, and all-day warming treatments*.

Sufficient and timely availability of irrigation water, complemented by a low relative humidity (RH), helps plants to maintain the plants' tissue temperature well below the critical threshold because of the efficient transpiration-mediated cooling (Weerakoon et al., 2008; Wassmann et al., 2009; Julia and Dingkuhn, 2013), which is an effective mechanism to cope with warming. However, a huge amount of rainfall in 2016 led to a higher air relative humidity in the anthesis stage (Figures 1E,F), which probably restricted the transpiration rate, causing a higher tissue temperature in the spikelets. Therefore, the higher tissue temperature in the spikelets resulted in high spikelet sterility, which may be another reason that the effect of the elevated temperature on yield performance in 2016 was much more severe than in 2015. Several previous studies have also recorded a significant reduction of the percent spikelet fertility in response to different temperature increases (Matsui et al., 1997a,b; Prasad et al., 2006a; Jagadish et al., 2007; Fahad et al., 2016b,c). The poor development as well as the impairment of the pollen grains can lead to decreased spikelet fertility (Zinn et al., 2010). During the flowering phase, both male and female gametophytes are sensitive, and the pollen grains are more sensitive than the ovules (Peet et al., 1998). In the present study, we examined the effects of three different elevated-temperature treatments, imposed for the entire duration of rice growth, including the sensitive reproductive stage, on the characteristics of the pollen grains of the cultivars. The results demonstrated that substantial pollen mortality caused by an increase of ~2°C led to spikelet sterility in both years, especially in 2016 (Figure 3). The pollen germination of the different genotypes was more variable at a higher temperature because of reduced pollen production due to anther indehiscence, poor pollen shedding and pollen interception by the stigma in a similar manner as reported by Jagadish et al. (2010). Poor anther dehiscence is mainly due to the tight closure of the locules, which results in less pollen dispersal. With respect to the number of pollen grains and germinated pollen on the stigma, our results clarified that due to the severity of the temperature increase in the microspore and anthesis phases, the pollen grains did not adhere to or germinate on the stigma, although those pollen grains were seemingly normal and stained well (Figure 4). Another reason for a decline in pollen germination may be poor pollen retention in the dehisced anthers. The pollen wall plays a key role in pollen germination on the stigma (Edlund et al., 2004). Numerous constituents of the pollen wall are delivered to the pollen surface by the tapetal cells, and the importance of the lipid components for pollen fertility has been recognized (Preuss et al., 1993; Hulskamp et al., 1995; Ariizumi et al., 2004). Interestingly, the pollen germination percentage was not impacted by the elevated-temperature treatments for any cultivars in 2015 or 2016 (Figures 4C,D), which indirectly indicates that the number of germinated pollen grains on the stigma was mainly related to the number of pollen grains adhering to the stigma. Therefore, understanding the mechanism leading to the lower quantity of pollen grains adhering to the stigma is the most useful and effective way to monitor the breeding of high-temperature-resistant varieties in the future.

Yield and its components, dry matter accumulation, biomass, and HI exhibited the greatest negative responses in the AW treatment in both 2015 and 2016. The significant reduction in the investigated attributes caused by the AW treatment resulted from the presence of an elevated temperature for the entire day (24 h), ensuring a comparatively greater rise in the cumulative temperature. This temperature increase might be a possible reason for the greater reduction in all of attributes recorded in the AW treatment. No differences in the yield were seen in the NW or DW treatment compared to the control in 2015, while in 2016, the yield in the NW treatment was reduced by 18–55%, which was approximately twice that of the grain yield decrease of 10% for each 1°C increase in the minimum temperature in the growing-season reported by Peng et al. (2004). Shah et al. (2014) and Chaturvedi et al. (2017) reported similar results, but Dong et al. (2011) reported that no significant difference or interaction in the actual rice growth response was found between warming regimes. These differences might be attributed to the interannual differences in the responses of rice to elevated temperature. Simultaneously, other environmental factors such as radiation, relative humidity and rainfall also play a vital role in the inconsistent results of different researches. However, the present study also found a reduction in grain yield of ~20–52% in the DW treatment in 2016, and there were significant reductions in the number of spikelets per m^2^ in the DW treatment in 2016 for HHZ, SY63 and LYPJ which was different from the results of Peng et al. (2004). We assume that this difference was mainly due to a larger increase in the DW treatment than used by Peng et al. (2004), who reported that annual mean maximum temperatures only increased by 0.35°C during the period between 1979 and 2003, which caused an inconspicuous effect of maximum temperature on crop yield.

Several stages comprise the entire duration of rice growth, and each stage has a characteristic threshold temperature above which significant adverse impacts are observed. However, the temperature fluctuation over the entire duration of rice growth is difficult to predict. The differences between the basic temperatures in different years and the differences in the times that rice encounters stressful temperatures will cause significantly the different rice production performance in different years. Li et al. (2003) showed that tiller production is a key agronomic trait in rice and is very sensitive to temperature, but no significant differences were seen in 2015 or in 2016 in the present study (data not presented). Our results show that the range of HI was 21.08–54.9% between 2015 and 2016 (Table 1), which was consistent with the results of Wu et al. (2016). The 43.5–54.9% HI in 2015 with an impact in the filling stage and the 21.1–45.1% HI in 2016 with impacts in the anthesis and filling stages illustrated that the high-temperature stress during the flowering period in 2016 induced the significant interannual differences in HI, because the temperature in 2015 was closed to the threshold temperature corresponding to the flowering period, whereas in 2016, it exceeded the threshold temperature, even though only an ~2°C temperature difference occurred between those 2years (Figure 1). This finding indicates that whether the anthesis was subject to temperature stress during the entire duration of rice growth determined the safety of rice production, a conclusion supported by Jagadish et al. (2007).

Genotypic variations in the plants' response to heat stress were evident among the different ecotypes. Shah et al. (2014) revealed that japonica ecotypes were much more sensitive to heat stress with respect to yield than indica ecotypes. In the present study, we used four indica ecotypes have been widely planted in the central and southern regions of China as our experimental materials to investigate their responses to elevated temperature. In terms of the constancy of yield and the performances of other parameters for the genotypes exposed to elevated temperature, cultivars SY63 and YLY6 are more tolerant than the other two genotypes, and LYPJ seemingly performs the worst. The percentages of the reduction in spikelets fertility of SY63 and YLY6 were less than those of HHZ and LYPJ, which illustrated that the differences in the heat resistance for the different genotypes were mainly determined by the responses of spikelets fertility to temperature. In a similar fashion, Peng et al. (2004) revealed that elevated temperatures primarily reduced the grain yield due to diminished spikelet fertility. Therefore, adopting reasonable agricultural management and effective methods to reduce the damage of high temperature during the flowering period will play an important roles in rice production in future climate warming scenarios.

SY63 is one of the most heat-tolerant hybrids for rice production in China. However, its complex three-line hybridization, non-ideal plant type and lower production potential has spurred the breeding of new genotypes with good comprehensive characteristics. The two-line hybrid rice LYPJ is a hallmark for improved rice breeding in the China National High-tech R&D Program. LYPJ has had the largest planting area for years after the widely planted three-line hybrid SY63 (LÜ and Zou, 2016). However, its breeding was accomplished with a photo-thermo-sensitive genic male sterile line, which sets the stage for spikelet sterility caused by higher temperature (Tables 3, 4). Zhao et al. (2006) suggested that YLY6 possesses a strong source, great sink activity and efficient flow, which lays a physiological basis for its high seed-setting rate and good grain filling. Our research revealed that these characteristics can be expressed even under elevated-temperature conditions. Therefore, additional research is needed to fully understand whether YLY6 can be planted as a heat-tolerant genotype in these climate change scenarios.

## Conclusion

In summary, the apparatus developed for heating the plots in this study was able to raise the air temperature in the canopy nearly 2°C above the control, and the elevated-temperature treatments affected the yield performance of all four rice cultivars in both years. However, there was a closed relationship between the reduction in grain yield caused by elevated temperature treatment and the climate conditions of a given year. The AW treatment had more deleterious effects than the DW and NW treatments because of a comparatively higher accumulated temperature. In the present study, the AW treatment reduced the yield in a range of 22–62% in both years compared to the control, and the reduction in 2016 was more severe than in 2015. The lowest grain yield in the AW treatment was associated with the spikelet filling percentage and grain weight due to the high-temperature stress. The reduction of yield in the NW treatment was similar to that reported in previous studies. However, the DW treatment significantly reduced the yield by reducing the number of spikelets per m^2^. The AW treatment accelerated the pollen mortality and affected the grain-filling process and storage, which in turn decreased the final grain yield. Of all the genotypes, SY63 and YLY6 had relatively higher tolerance to heat stress. The above results indicate that further field studies are required to reveal the mechanism leading to the reduced number of pollen grains adhering to the stigma.

## Author contributions

JH conceived the idea of the experiment. ZY and ZZ had major and equal contribution in overall preparation. TZ and SF gathered the literature and contributed in arranging the data for different attributes. SF, KC, LN, SP, and JH provided the technical guidance and editing support.

### Conflict of interest statement

The authors declare that the research was conducted in the absence of any commercial or financial relationships that could be construed as a potential conflict of interest.
