# Supplementary material for: The Effect of Season-Long Temperature Increases on Rice Cultivars Grown in the Central and Southern Regions of China
Source: Front Plant Sci. 2017 Nov 6;8:1908. doi: 10.3389/fpls.2017.01908 (PMC5682563; doi:10.3389/fpls.2017.01908)
Supplement: Supplementary file 1 [file DataSheet1.docx]

**
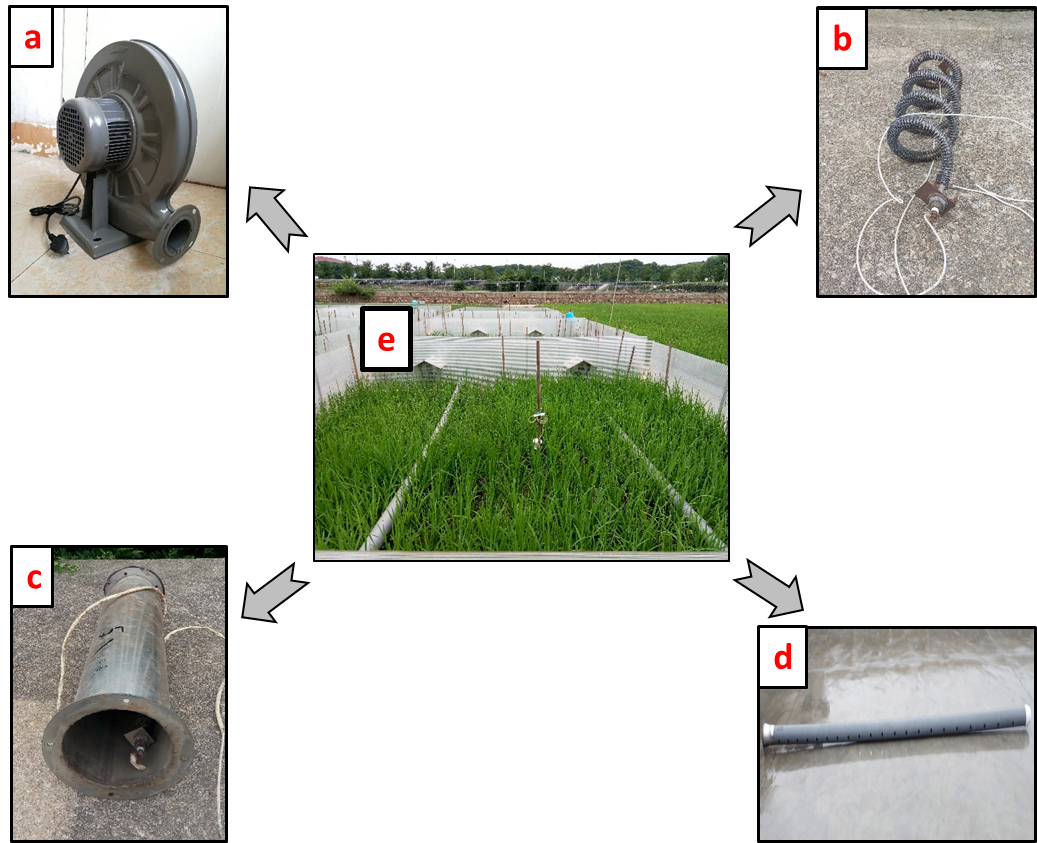
Supplementary Figures**

**Fig. 1. The structure of the open-top hot-blast system.**

(a) Blower used to push warming air. (b) Heater which was placed in front of the blower to heat the air. (c) Iron covering protecting the heater and directing the heat. (d) PVC pipes with an inner diameter of 11 cm used to transfer the heated air by the non- uniform distribution of round holes. (e) Sunshine plate used to keep the main plots at a constant temperature during the entire duration of rice growth.


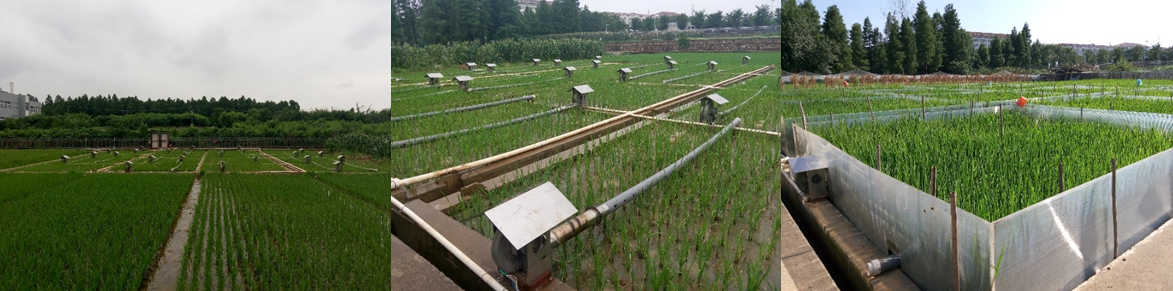


**Fig. 2. All the pictures of the plot arrangements and the heating apparatus are from the field experiment**

| **Block 4 Block 3 Block 2 Block 1** | | | | | | | | |  |
| --- | --- | --- | --- | --- | --- | --- | --- | --- | --- |
| V1  CK | V3 | V2 | | V4 | V4 | V2 | V3 | V1 | **Ditch** |
| V2 | V4 | V1 | | AW  V3 | V3 | DW  V1 | AW  V4 | V2 |  |
| Ditch | | | | | | | | |  |
| V3 | V1 | V4  NW | | V2 | V1 | V3 | V2 | V3 |  |
| V2 | NW  V4 | V3 | | V1 | V4 | CK  V2 | V1 | DWW  V4 |  |
| V4 | V2  DWDW | V1 | | V3 | V4 | V1 | V3 | V2 |  |
| V1 | V3 | V4 | | CK  V2 | V2 | AW  V3 | V4 | NW  V1 |  |
| Ditch | | | | | | | | |  |
| V4  AWW | V2 | V4 | | V1 | V2 | V3 | V1 | V4 |  |
| V3 | V1 | V2 | | DWDW  V3 | V1 | NW  V4 | V2 | CK  V3 |  |
| **Design:**  Split plot with 4 blocks  **Transplanting：**  16.7 cm × 20.0 cm | | | **Temperature management:**  DW ( Daytime warming)  NW ( Night-time warming)  AW ( All-day warming)  CK ( Ambient control) | | | **Varieties:**  V1 = HHZ  V2 = LYP9  V3 = XY63  V4 = YLY6 | | **N**  **E** | |

**Fig. 3. Plot design map**
